# Clinical characteristics and outcomes of COVID-19 in pediatric patients with rheumatic diseases

**DOI:** 10.1038/s41390-024-03561-1

**Published:** 2024-10-07

**Authors:** Yating Wang, Shu Su, Mingsheng Ma, Ruohang Weng, Zhiyong Zhang, Dawei Liu, Xin Yan, Junjun Wang, Yajun Wang, Wei Zhang, Sirui Yang, Hongxia Zhang, Dongmei Zhao, Meiping Lu, Xiaoqing Li, Jia Zhu, Weixi Zhang, Haiguo Yu, Dongfeng Zhang, Yanjie Huang, Guangmin Nong, Xuxu Cai, Huawei Mao, Fei Sun, Xiaochuan Wu, Zanhua Rong, Jianjiang Zhang, Zhixiang Li, Xinhui Jiang, Xiaozhong Li, Xuemei Liu, Chongwei Li, Lifeng Sun, Sihao Gao, Jun Yang, Hongmei Song, Xuemei Tang, Chinese Alliance of Pediatric Rheumatic and Immunologic Diseases

**Affiliations:** 1 Department of Rheumatology and Immunology Children’s Hospital of Chongqing Medical University, National Clinical Research Center for Child Health and Disorders, Ministry of Education Key Laboratory of Child Development and Disorders. Chongqing Key Laboratory of Child Rare Diseases in Infection and Immunity, Chongqing, China; 2 Department of Epidemiology and Biostatistics, The Second Affiliated Hospital of Chongqing Medical University, Chongqing, China; 3 Department of Pediatrics, Peking Union Medical College Hospital, Chinese Academy of Medical Sciences, Peking Union Medical College, Beijing, China; 4 Rheumatology & Immunology Department of Shenzhen Children’s Hospital, Shenzhen, China; 5 Department of Rheumatology and Immunology, The First People’s Hospital of Yunnan Province, Kunming, China; 6 Pediatric Immunology and Rheumatology Department, Chengdu Women’s and Children’s Central Hospital, School of Medicine, University of Electronic Science and Technology of China, Chengdu, 611731 China; 7 Department of pediatric rheumatology, immunology, and allergy, The First Hospital of Jilin University, Changchun, Jilin China; 8 Department of Pediatric Nephrology, Rheumatology and Immunology, Children’s Hospital Affiliated to Shandong University, Jinan Children’s Hospital, Jinan, Shandong China; 9 Department of Rheumatology and Immunology, Children’s Hospital of Urumqi, No.1 Jiankang Rd., TianShan Distinct, Urumqi, China; 10 Department of Rheumatology Immunology and Allergy, Children’s Hospital, Zhejiang University School of Medicine, National Clinical Research Center for Child Health, Hangzhou, China; 11 Department of Rheumatology and Immunology, Xi’an Children’s Hospital, Xi’an, 710068 China; 12 Department of Rheumatology and Immunology, The Affiliated Children’s Hospital, Capital Institute of Pediatrics, 2 Yabao Road, Chaoyang District, 100020 Beijing, China; 13 Department of Pediatric Allergy and Immunology, The Second Affiliated Hospital and Yuying Children’s Hospital of Wenzhou Medical University, Wenzhou, 325027 China; 14 Department of Rheumatology and Immunology, Children’s Hospital of Nanjing Medical University No. 72 Guangzhou Road, Nanjing, 210008 Jiangsu China; 15 Department of Pediatric Nephrology, Children’s Hospital of Hebei Province affiliated to Hebei Medical University, Shijiazhuang, China; 16 Department of Pediatrics, The First Affiliated Hospital of Henan University of Chinese Medicine, Zhengzhou, 450046 China; 17 Department of Pediatrics, The First Affiliated Hospital of Guangxi Medical University, Nanning, 530021 China; 18 Department of Pediatrics, Shengjing Hospital of China Medical University, Shenyang, 110004 Liaoning PR China; 19 Department of Immunology, Ministry of Education Key Laboratory of Major Diseases in Children, Beijing Children’s Hospital, National Center for Children’s Health, Capital Medical University, No. 56 Nanlishi Road, 100045 Beijing, China; 20 Department of Pediatrics, The Second Xiangya Hospital, Central South University, Changsha, China; 21 Department of paediatrics, The Second Hospital of Hebei Medical University, Shijiazhuang, China; 22 Department of paediatrics, The First Affiliated Hospital of Zhengzhou University, Zhengzhou, 450052 China; 23 Inner Mongolia Autonomous Region People’s Hospital, Hohhot, China; 24 Guiyang Maternal and Child Health Care Hospital (Guiyang Children’s Hospital), Guiyang, China; 25 Department of Nephrology and Immunology, Children’s Hospital of Soochow University, Suzhou, China; 26 Qilu Children’s Hospital of Shandong University, Jinan, China; 27 Department of Rheumatology & Immunology, Tianjin Children’s Hospital, No. 238 Longyan Road, Beichen District, Tianjin, China; 28 Department of Pediatrics, Provincial Hospital Affiliated to Shandong University, Jinan, 250021 China

## Abstract

**Background:**

This study investigates the clinical characteristics and outcomes of pediatric patients with rheumatic diseases infected with COVID-19 in China.

**Methods:**

We conducted a retrospective analysis of pediatric patients with rheumatic diseases who contracted COVID-19. Data were collected via a comprehensive questionnaire with a 14-day follow-up. Multivariable logistic regression was used to assess severe outcomes, and network analyses evaluated symptom correlations.

**Results:**

A total of 1070 cases were collected. Fever (88.05%) and cough (62.75%) were the most common symptoms. Cough, nasal congestion, and runny nose exhibited a stronger correlation with each other. A higher incidence of fever reduced the incidence of two single symptoms (nasal congestion [*r* = −0.833], runny nose [*r* = −0.762]). Vaccinated children showed a shorter time to negative COVID-19 conversion (7.21 days vs. 7.63 days, *p* < 0.05) and lower hospitalization rates (*p* = 0.025). Prolonged symptom duration was associated with older age (OR: 1.07 [1.04–1.11]; *p* < 0.001) and systemic lupus erythematosus (OR: 1.47 [1.01–2.12]; *p* = 0.046).

**Conclusions:**

Pediatric patients with rheumatic diseases exhibited a wide range of clinical symptoms after COVID-19 infection. The infection generally did not lead to severe outcomes in this study. COVID-19 vaccination was associated with reduced hospitalization risk and expediting the time to negativity for virus.

**Impacts:**

This manuscript demonstrates a comprehensive analysis of the clinical characteristics and outcomes of COVID-19 infection in pediatric patients with rheumatic diseases in China. It provides critical insights into the specific challenges faced by this vulnerable population and offers practical recommendations for improving patient management during periods of increased infectious risk.

## Introduction

Since its emergence in January 2020, COVID-19 has rapidly evolved into a global public health crisis, significantly impacting worldwide health systems and societies worldwide. The virus has resulted in millions of confirmed cases and fatalities globally.^1,2^ The high transmissibility of COVID-19, compounded by the continuous emergence of new variants, has intensified the social and medical challenges posed by the disease, particularly among vulnerable populations such as children, who appear to have a heightened susceptibility.^3,4^

Children, especially those with rheumatic diseases, are at an elevated risk of contracting COVID-19 due to both the dysregulation of their immune systems and the immunosuppressive treatments used for managing their underlying conditions.^5^ These factors can increase their vulnerability to infections. Evidence from previous studies in Hubei on adults indicates that patients with rheumatic diseases may have a higher susceptibility to COVID-19 infection compared to the general population.^6^ Moreover, COVID-19 can cause excessive immune activation leading to immune damage to vital organs. The administration of corticosteroids and other immunosuppressants in severe cases may further exacerbate the risk of infection. Previous studies have found that common manifestations of COVID-19 in pediatric and adult patients with rheumatic diseases include fever, cough, and fatigue, with a small proportion progressing to pneumonia and some requiring intensive care.^7,8^ However, these studies were primarily conducted abroad, and it is known that ethnicity may influence COVID-19 outcomes. For instance, Asians may be at a higher risk of requiring intensive care unit admission and experiencing higher mortality rates compared to other populations.^9^ Consequently, there is a pressing need to conduct a large-scale, national study on the clinical features and outcomes of COVID-19 among pediatric patients with rheumatic diseases in China.

Comprehending the clinical features and outcomes of COVID-19 in pediatric patients with rheumatic diseases is essential for the effective prevention and management of the disease in this vulnerable population. This study aims to investigate the clinical characteristics and outcomes of pediatric patients with rheumatic diseases who are infected with COVID-19 through a national multicenter cohort study in China.

## Methods

### Study design

With reference to the clinical characteristics related to infection with the COVID-19 Omicron strain in China, we developed a comprehensive questionnaire that included a range of relevant demographic and clinical factors and outcomes. These factors included age, sex, residence, primary disorders, current medication use, date and basis of COVID-19 infection diagnosis, route of infection, vaccination status. Daily symptoms for each day during the first 14 days post-COVID-19 infection or until full recovery, whichever occurred first. Symptoms included fever, sore throat, weakness, muscle aches, abdominal pain, diarrhea, vomiting, cough, runny nose, nasal congestion, irritability, loss of taste, asymptomatic status, and other symptoms. The duration until a negative nucleic acid test result was collected. The questionnaire was administered through a web-based platform and disseminated through various channels, including outpatient clinics, inpatient wards, patient support groups, and social media. Participants meeting the study’s eligibility criteria voluntarily completed the questionnaire, with data collection and data curation carried out by study physicians.

### Inclusion and exclusion criteria

The inclusion criteria were as follows: (i) age <18 years; (ii) primary rheumatic diseases and primary immunodeficiency diseases (including juvenile idiopathic arthritis, systemic lupus erythematosus, Henoch-Schonlein purpura, Kawasaki disease, aortitis, primary immunodeficiency, juvenile dermatomyositis, etc.) that had been diagnosed or were being followed up; and (iii) COVID-19 nucleic acid test or antigen positive;(iv) willingness to participate. Participants were excluded from this study if they had incomplete demographic information such as sex, date of birth, or current disease in the questionnaire, if they had not self-reported their symptoms more than three times or if they had obvious error on disease progression within 14 days.

### Participant recruitment

From December 26, 2022, to January 31, 2023, a total of 1070 valid cases were collected to investigate the symptoms of COVID-19 infection in pediatric patients with rheumatic diseases.

### Ethics statement

This study has been approved by the Institutional Review Board of Children’s Hospital of Chongqing Medical University (File No:202337/Date:February 17,2023).The waiver of participants’ consent was obtained given that all the participants in the cohort are de-identified, and the study design is totally observed, written informed consent for participation was not required for this study.

### Statistical analysis

Appropriate statistical methods were applied to describe the quantitative and qualitative variables of the study population. For quantitative variables that followed a normal distribution, the means and standard deviations were reported, and a t test for independent samples was used to compare groups. For quantitative variables that did not follow a normal distribution, the M (P25, P75) was reported, and a rank sum test was performed for comparisons. Qualitative variables were presented as case counts and percentages, and a chi-square test was performed for comparisons. Univariate logistic regression analysis was used to assess the relationship between vaccination, duration of COVID-19 symptoms, and covariates. The results were expressed as odds ratios (OR) with 95% confidence intervals (CI). Statistical significance was defined as *P* < 0.05.

Thirteen individual symptoms and the top 10 symptom combinations within 14 days were collected and presented using R software (version 4.1.3). The evolution of symptom proportions and status changes over time was presented. A Sankey diagram was constructed to illustrate transitions from one symptom to another within 14 days. Pearson correlation analyses were conducted using R software (version 4.1.3) to calculate the relationships between symptoms. Significant correlations (*P* ≤ 0.05) between symptom pairs were identified, and a heatmap was employed to visualize the correlation results.

## Results

We collected a total of 1070 valid cases, comprising 448 males (41.86%) and 622 females (58.13%), resulting in a male-to-female ratio of 1:1.39. The mean age of the participants was 10.3 ± 4.53 years, with an age range spanning from 3 months to 17 years. The primary disorders included juvenile idiopathic arthritis (430, 40.19%), systemic lupus erythematosus (265, 24.77%), juvenile dermatomyositis (59, 5.51%),Henoch-Schonlein purpura (55,5.15%),primary immunodeficiency (50, 4.67%) and other rheumatic diseases. A total of 463 (43.27%) people were treated with corticosteroids and 487 (45.51%) patients were treated with biologic disease-modifying anti-rheumatic drug (bDMARDs). Regarding vaccination status, 459 participants (42.90%) had received the COVID-19 vaccine, while 611 (57.10%) had not. Most of the patients had good prognosis; 45 (4.21%) participants were hospitalized for COVID-19, of whom 12 were diagnosed with upper respiratory infections, 10 with bronchitis, 10 with pneumonia, 2 with multiple system inflammatory syndrome (MIS-C), and 11 with other diagnoses. No deaths were reported (Table 1). Participants were distributed across various regions of China, with the highest numbers from Sichuan (115 cases), Shandong (103 cases), Guangdong (94 cases), and Chongqing (75 cases). Analysis of the timeline of COVID-19 diagnoses indicated that the peak of confirmed cases occurred between December 17 and December 23, 2022, within the two-week period between December 12 and December 25, 2022.

**Table 1:** Demographic characteristics

			Cases (*N* = 1070)
Sex			
	Male		448 (41.86%)
	Female		622 (58.13%)
Age			
	0–6 years		268 (25.05%)
	7–12 years		427 (39.91%)
	13–17 years		375 (35.04%)
Primary disorders			
	Juvenile idiopathic arthritis		430 (40.19%)
	Systemic lupus erythematosus		265 (24.77%)
	Juvenile dermatomyositis		59 (5.51%)
	Henoch-Schonlein purpura		55 (5.14%)
	Primary immunodeficiency		50 (4.67%)
	Aortitis		48 (4.48%)
	Autoinflammatory diseases		48 (4.48%)
	Kawasaki disease		34 (3.18%)
	Sjogren’s syndrome		24 (2.24%)
	Behcet’s disease		11 (1.03%)
	Scleroderma/Systemic sclerosis		6 (0.56%)
	Others^b^		40 (3.74%)
Regimen			
	Corticosteroids		
		Prednisone acetate	432 (40.37%)
		Methylprednisolone	31 (2.90%)
	bDMARDs^a^		487 (45.51%)
Route of infection			
	Exposure to family infection		686 (64.11%)
	Outbreak of infection		129 (12.06%)
	Not sure		255 (23.83%)
Novel coronavirus vaccination status			
	Vaccinated		459 (42.90%)
	Not vaccinated		611 (57.10%)
Hospitalization			
	Total		45 (4.21%)
	Upper respiratory infections		12 (1.12%)
	Bronchitis		10 (0.93%)
	Pneumonia		10 (0.93%)
	Multiple system inflammatory syndrome (MIS-C)		2 (0.19%)
	Other diagnose		11 (1.0%)

^a^bDMARDs included golimumab, adalimumab, infliximab, recombinant human type II tumor necrosis factor receptor antibody fusion protein for injection, tetrasip, belimumab, abciximab, anabolic acid, ranibizumab, etc.

^b^Others include ANCA-associated vasculitis, polyarteritis nodosa, mixed connective tissue disease, etc.

Of the 1070 participants, fever was the most frequently reported symptom (943 cases, 88.05%), followed by cough (672 cases, 62.75%). Other common symptoms included sore throat (409, 38.19%), weakness (397, 37.07%), runny nose (390, 36.41%), and nasal congestion (362, 33.80%). Table 2 provides a detailed breakdown of the number of cases and prevalence of each symptom.

**Table 2:** Major clinical manifestations after COVID-19 infection in children with rheumatic immune disease

Symptoms	Number of cases (*N* = 1070)	Prevalence (%)	Duration [M (P25, P50, P75), d]
Fever	943	88.05	2.15 (1.0,2.0,3.0)
Cough	672	62.75	5.19 (2.0,4.5,7.0)
Sore throat	409	38.19	3.14 (1.0,2.0,4.0)
Weakness	397	37.07	3.20 (1.0,2.0,4.0)
Runny nose	390	36.41	4.34 (2.0,3.0,6.0)
Nasal congestion	362	33.80	4.05 (2.0,3.0,6.0)
Muscle soreness	306	28.57	2.51 (1.0,2.0,3.0)
Vomiting	151	14.10	1.45 (1.0,1.0,2.0)
Hypogeusia	120	11.20	3.68 (1.0,3.0,5.0)
Diarrhea	101	9.43	1.81 (1.0,1.0,2.0)
Abdominal pain	83	7.75	2.13 (1.0,2.0,3.0)
Fidgeting	79	7.38	2.89 (1.0,2.0,4.0)
No symptoms	23	2.15	/

**Fig. 1:**
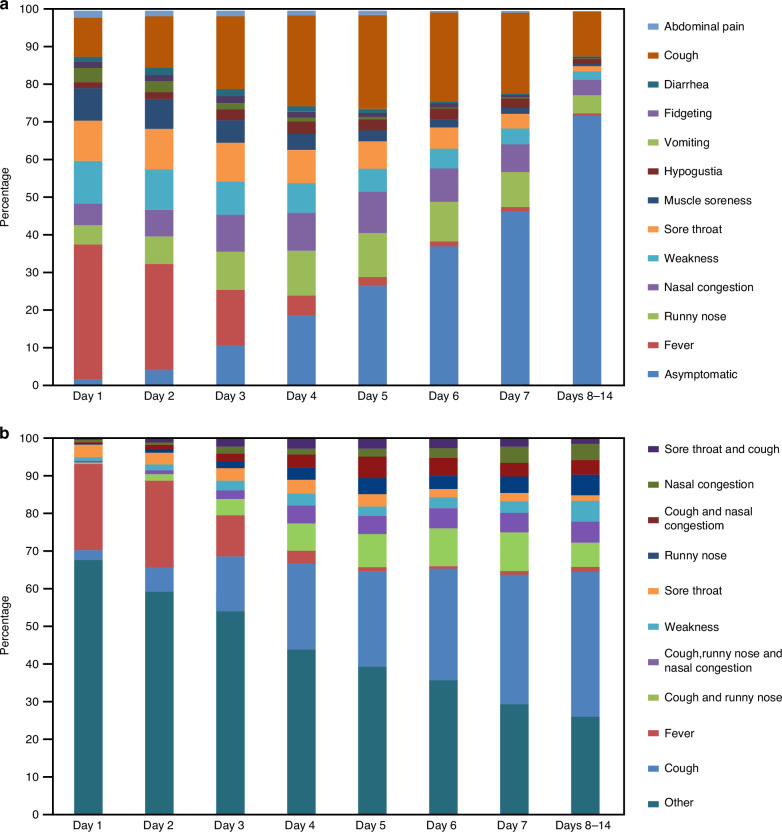
Temporal distribution of individual symptom and symptom combinations in patients following COVID-19 infection over a 14-day period. **a** Temporal Trends and Composition of Each Symptom Post-COVID-19 Infection. Description: This figure illustrates the temporal distribution and proportion of individual symptoms over the course of COVID-19 infection. Early-stage symptoms prominently include fever, sore throat, muscle soreness, and weakness. As the disease progresses into the middle and later stages, cough, runny nose, and nasal congestion become more prevalent. The incidence of asymptomatic cases increases progressively over time. **b** Temporal Trends and Incidence of the Top 10 Symptoms and Symptom Combinations. Description: This figure showcases the temporal trends and incidence rates of the top 10 individual symptoms and their combinations. “Other” represents symptoms that are not among the top 10 or combinations thereof. This categorization helps in understanding the shifting symptomatology and the overall impact of COVID-19 on pediatric patients with rheumatic diseases.

The temporal evolution and composition of symptoms revealed that fever, sore throat, muscle aches, and weakness were the predominant symptoms during the first 1–3 days of the disease course. Subsequently, the proportion of fever, vomiting, and muscle soreness declined gradually, while symptoms such as cough, runny nose, nasal congestion, and fidgeting increased gradually, peaking on days 4–5 of the disease course and then declining gradually. After the 7th day of illness, over 40% of the pediatric patients experienced symptom resolution (Fig. 1a). The top 10 symptoms and symptom combinations during the first 7 and 8–14 days of the disease course were cough, fever, cough + runny nose, cough + runny nose + nasal congestion, weakness, sore throat, runny nose, cough + nasal congestion, nasal congestion, and sore throat + cough. The trends of these symptoms over the course of the disease are depicted in Fig. 1b.

**Fig. 2:**
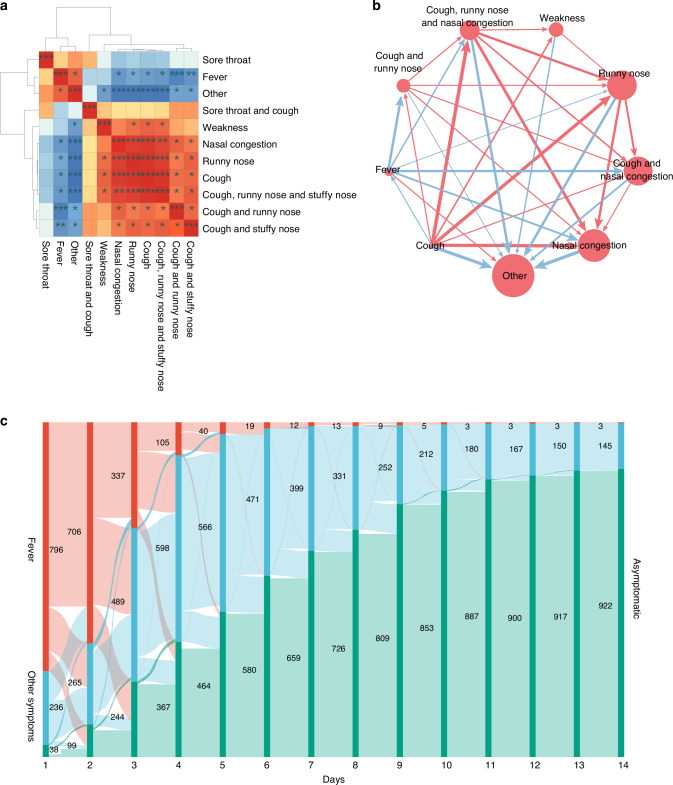
Correlation analysis and temporal trends of key symptoms and symptom combinations. **a** Association Among the Top 10 Clinical Symptoms and Symptom Combinations by Prevalence. Description: This figure illustrates the relationships between the top 10 most common symptoms and their combinations, highlighting positive correlations among cough, runny nose, and nasal congestion. These symptoms are inversely correlated with the incidence of fever, suggesting that as one set of symptoms increases, the likelihood of fever decreases. **b** Correlation Analysis Between Clinical Symptoms and Their Combinations. Description: This figure provides a detailed correlation analysis of the incidence rates of various symptoms and their combinations. It identifies significant associations and trends, helping to understand how symptoms tend to cluster together or occur independently. **c** Temporal Changes in Fever, Other Symptoms, and Asymptomatic Status Over Time. Description: This figure depicts the dynamic changes in the prevalence of fever, other symptoms, and asymptomatic status throughout the course of the disease. Fever decreases steadily over time, while the incidence of non-fever symptoms increases during the early stages, peaking around days 4–5, and then gradually diminishes. The proportion of asymptomatic cases rises progressively as the disease progresses.

Correlation analysis revealed that cough, nasal congestion, runny nose, and weakness were positively correlated and tended to occur simultaneously. Cough, nasal congestion, and runny nose exhibited a stronger correlation with each other than with fever, which was negatively correlated with the aforementioned symptoms. A higher incidence of fever reduced the incidence of two single symptoms (nasal congestion [*r* = −0.833] and runny nose [*r* = −0.762]) and three sets of combined symptoms (cough and runny nose [*r* = −0.929], cough and nasal congestion [*r* = −0.857], and cough, runny nose, and nasal congestion [*r* = −0.833]) and was reduced by cough (*r* = −0.810). Moreover, nearly all symptoms, except fever (*r* = 0.810), reduced the incidence of non-top 10 symptoms (ranging from −0.762 to −1.000) (Fig. 2a, b). The trends in fever, symptoms other than fever, and no symptoms over 14 days are visualized in the Sankey diagram (Fig. 2c).

A total of 445 cases had negative COVID-19 nucleic acid or antigen conversion at the 14^th^ day of questionnaire completion, with a mean conversion time of 7.40 ± 2.23 days. Pediatric patients who received the COVID-19 vaccine had a shorter time to negative conversion than those who did not receive the vaccine (7.21 days vs. 7.63 days, *P* < 0.05). Among the medication groups, the time to negative COVID-19 conversion was shorter in the prednisone acetate (5 mg/d < dosage < 20 mg/d)/methylprednisolone (4 mg/d < dosage < 16 mg/d) group than in the no corticosteroid group (7.01 days vs. 7.73 days, *P* < 0.05). The prednisone acetate (≥20 mg/d)/methylprednisolone (≥16 mg/d) group had an even shorter time to COVID-19 negativity (6.83 days vs. 7.73 days, *P* < 0.05). The use of bDMARDs did not have a significant effect on the time to negative COVID-19 conversion (*P* > 0.05) (Table 3).Our findings indicate that hospitalization rates in the vaccinated group were significantly lower compared to the unvaccinated group (*p* = 0.025). However, no difference was observed in the duration of symptoms based on vaccination status (Not vaccinated 6.87 ± 2.85 vs. vaccinated 6.78 ± 2.83, *P* > 0.05) (Appendix Table S1). We conducted a univariate logistic regression analysis to explore the relationship between COVID-19 vaccination status (unvaccinated, 1 dose, 2 doses, 3 doses) and the likelihood of hospitalization, as well as the duration of symptoms using 3-day and 7-day thresholds. The data indicated that receiving two doses of the vaccine was associated with a reduced risk of hospitalization (OR: 0.40 [95% CI: 0.17–0.83]; *P* = 0.021). In the analysis using a 3-day symptom threshold, receiving three doses of the vaccine was identified as a protective factor (OR: 0.34 [95% CI: 0.15–0.82]; *P* = 0.010). However, when using a 7-day symptom threshold, there was no significant difference in outcomes between different vaccine doses and no vaccination (Supplementary Appendix Table S2).

**Table 3:** Comparison of COVID-19 negative conversion times across vaccination, corticosteroid, and biologic groups.

Group		Number of cases	Time to negativity [M (P25, P75), d]	*P* value
COVID-19 vaccination	Not vaccinated	242	7.63 (5.75,9.0)	0.024
	Vaccinated	203	7.21 (5.0,8.0)	
Prednisone acetate/ Methylprednisolone (mg/d)	Not used	249	7.73 (6.0,9.5)^b,c^	0.014
	Dosage ≤5 (dosage ≤4)^a^	86	7.20 (5.0,9.5)	
	5 < dosage < 20 (4 < dosage < 16)^a^	80	7.01 (5.0,8.0)^b^	
	Dosage ≥20 (dosage ≥16)^a^	30	6.83 (5.0,8.25)^c^	
bDMARDs	Not used	247	7.42 (6.0,8.0)	0.808
	Used	198	7.47 (5.0,9.0)	

^a^Methylprednisolone dose.

^b^Difference in time to COVID-19 conversion compared to the prednisone acetate (5 < dosage < 20)/methylprednisolone (4 < dosage < 16) group in the no hormone group.

^c^Difference in time to COVID-19 conversion compared to the prednisone acetate (≥20 mg/d)/methylprednisolone (≥16 mg/d) group in the no corticosteroids group.

**Fig. 3:**
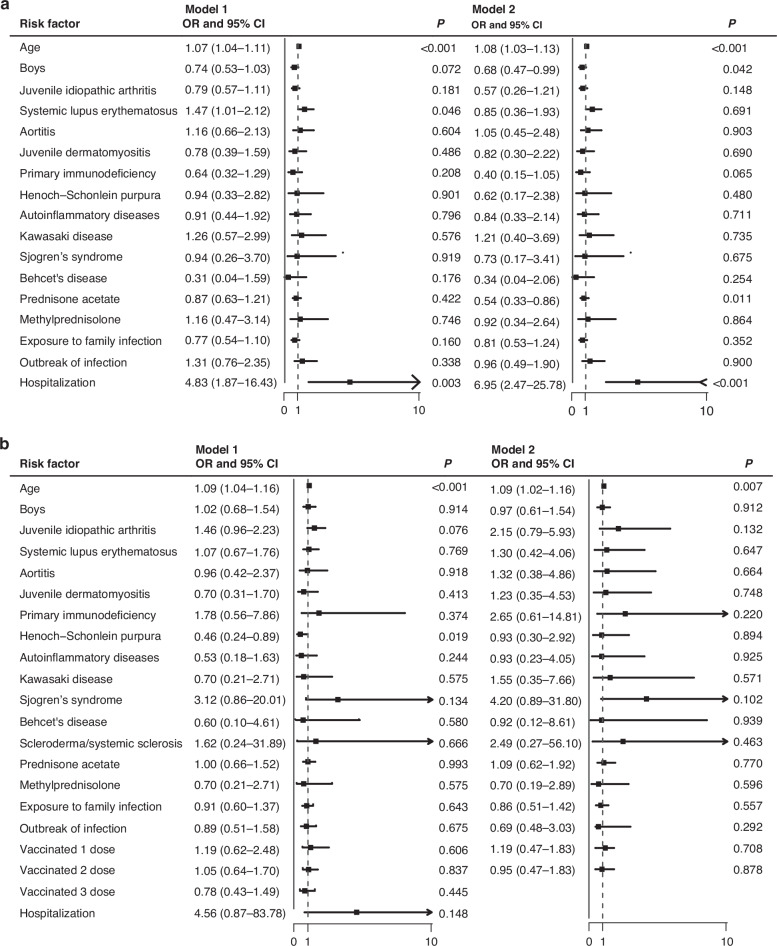
Attribution analysis of symptom duration using 3-day thresholds. Description: This set of figures illustrates the results of the attribution analysis for symptom duration using 3-days thresholds. **a** Displays the 3-day symptom duration threshold analysis for the unvaccinated group. It identifies the factors contributing to longer symptom durations in unvaccinated patients. **b** Shows the 3-day symptom duration threshold analysis for the vaccinated group. This figure highlights the impact of vaccination on reducing the duration of symptoms within this shorter timeframe.

**Fig. 4:**
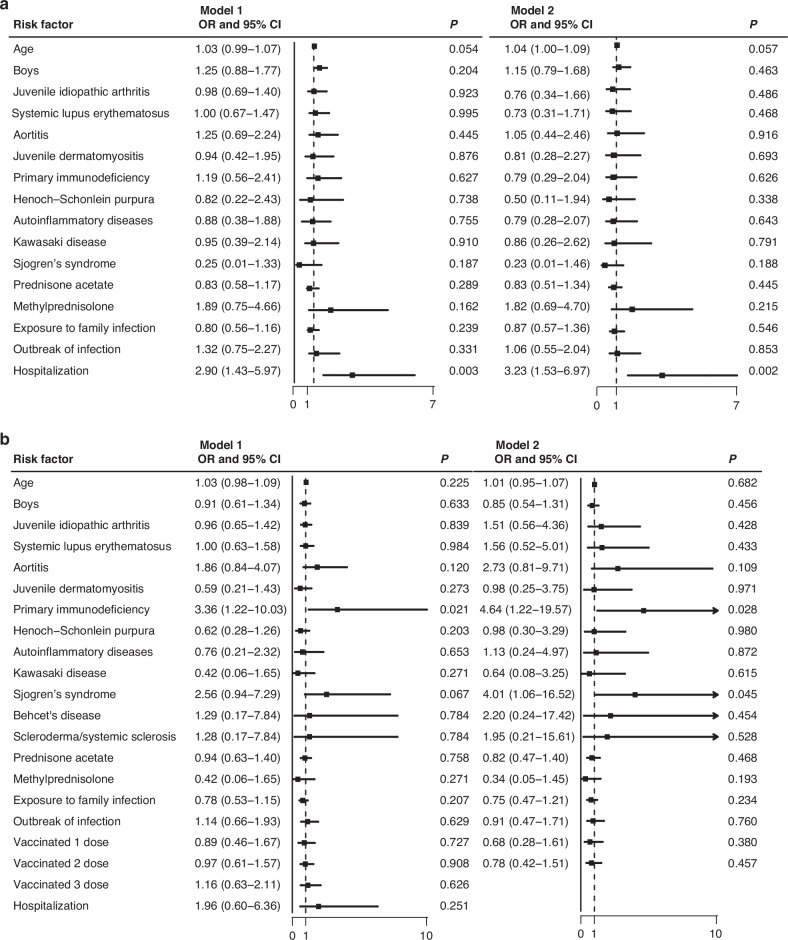
Attribution analysis of symptom duration using 7-day thresholds. Description: This set of figures illustrates the results of the attribution analysis for symptom duration using 7-days thresholds. **a** Represents the 7-day symptom duration threshold analysis for the unvaccinated group, revealing the contributing factors for prolonged symptoms in the absence of vaccination over a longer period. **b** Illustrates the 7-day symptom duration threshold analysis for the vaccinated group, providing insights into the influence of vaccination on mitigating prolonged symptoms in a more extended period.

The regression analysis utilized a 3-day and 7-day threshold for long symptom duration and categorized cases into a vaccinated group and an unvaccinated group, respectively. Model 1 presented the results from univariate logistic regression analysis, while Model2 displayed the results from multivariate logistic analysis. In 3-day model, in addition to the evident factor of prolonged symptom duration in hospitalized pediatric patients, our data showed that in the unvaccinated group, elder pediatric patients (OR: 1.07 [1.04–1.11]; *p* < 0.001) and those with systemic lupus erythematosus (OR: 1.47 [1.01–2.12]; *p* = 0.046) generally had longer durations. In the multivariate analysis, younger age (OR: 1.08 [1.03–1.13]; *p* < 0.001), male (OR: 0.68 [0.47–0.99]; *p* = 0.042) and prednisone use (OR: 0.54 [0.33–0.95]; *p* = 0.011)were associated with shorter symptom durations. In the vaccinated group, younger age (OR: 1.09 [1.04–1.06]; *p* < 0.001) and Henoch-Schonlein purpura (OR: 0.46 [0.24–0.89]; *p* = 0.019) were linked to shorter symptom durations. In the two groups, factors such as rheumatic diseases, glucocorticoid usage, and the route of infection were not associated with symptom duration (Fig. 3a, b). When a 7-day threshold was set for long symptom duration, in the unvaccinated group, patients with primary immunodeficiency disease (OR: 4.46 [1.22–19.57]; *p* = 0.028) and systemic sclerosis (OR: 4.01 [1.06–16.52]; *p* = 0.045) were identified as factors contributing to a longer duration of symptoms.In the vaccinated group, age, sex, type of disease, glucocorticoid usage, and route of infection were not found to be associated with symptom duration (Fig. 4a, b).

## Discussion

To our knowledge, this study is the first to comprehensively track the clinical symptoms within two weeks of COVID-19 infection in pediatric patients with rheumatic diseases in China. Our findings indicated that fever typically appeared within the first three days and was significantly negatively associated with all other symptoms and coughing persisted longer than other symptoms. Notably, vaccination was found to lower hospitalization risk and shorten the duration of negative conversion. Majority of participants had mild COVID-19 symptoms. Our results underscore the importance of monitoring a range of clinical symptoms that may positively correlate during the course of COVID-19 infection.

This study identified that fever, cough, runny nose, sore throat and other symptoms of upper respiratory tract infection as the most common manifestations of COVID-19 in pediatric patients during the epidemic period. These findings are consistent with a study conducted in New York on pediatric patients with rheumatic diseases, which also reported fever, fatigue, and cough as prevalent symptoms.^10^ Additionally, our results align with previous studies conducted on healthy children.^11^ Additionally, gastrointestinal symptoms, malaise, and irritability were observed, although they were less common and often not associated with the lower respiratory tract.

Regarding the correlation of symptoms, fever was the most prominent early symptom and typically the first to appear. As fever subsided, symptoms such as cough, nasal congestion, and runny nose became more prevalent, with cough being the longest-lasting symptom. The combination of cough and runny nose was the most frequent symptom pairing. Therefore, it is important to focus on fever in the initial 1–3 days of infection and on respiratory symptoms, which become more common after the fourth day. Prolonged cough may indicate potential lower respiratory tract involvement. A study from South Africa also found that fever (46%) and cough (40%) were the main symptoms in children infected with the Omicron variant.^12^ The prevalence of fever and cough in our study was significantly higher than in the South African study and previous reports,^13,14^ likely due to differences in virus strains. While loss of smell and taste was common in earlier COVID-19 infections, the prevalence of these symptoms decreased with the Omicron variant, with only 11.2% of children in our survey reporting loss of taste. In our cohort, approximately 2% of children were asymptomatic, a lower rate compared to the 12.9–38.9% reported in Western studies.^8,15^ This discrepancy may be due to differences in ethnicity, increased symptom burden from underlying rheumatic diseases, or the timing of nucleic acid or antigen testing, which may have missed asymptomatic cases. This discrepancy may be due to the difference in ethnics, increased symptom burden from underlying diseases or the timing of nucleic acid or antigen testing, which may have missed asymptomatic cases.

Pediatric patients with rheumatic diseases often receive immunosuppressive therapy, making them more susceptible to infections. However, previous research has shown that patients with rheumatoid arthritis or spondy loarthritis on biologic agents did not develop severe respiratory complications from COVID-19, suggesting these treatments may mitigate the abnormal inflammatory and cytokine responses that lead to severe disease.^16^ A similar study from Spain has suggested that treatment with glucocorticoids increases the risk of hospital admission due to COVID-19.^15^ Additionally, pediatric patients with childhood-onset systemic lupus erythematosus who are treated with rituximab therapy may face an increased risk of severe infections.^17^ A multicenter study involving 113 children with rheumatic diseases treated with multiple biologic disease-modifying antirheumatic drugs (bDMARDs) found no association between biologic therapy and adverse COVID-19 outcomes.^18^ Similarly, in our study, 45.51% of children were on bDMARDs, and we found that bDMARD use did not prolong the time to COVID-19 negativity. Conversely, corticosteroid use, including prednisone acetate (≥ 5 mg/d) and methylprednisolone (≥ 4 mg/d), was associated with a shorter time to COVID-19 negativity, likely due to reduced inflammatory activity, contrasting with previous findings that corticosteroids may prolong viral clearance.^19^ A Japanese study indicated that children with rheumatic diseases treated with traditional disease-modifying antirheumatic drugs (tDMARDs) did not show an elevated risk of contracting COVID-19 or experiencing severe outcomes from the disease.^20^ These data suggest that tDMARDs should be continued for these patients during a pandemic.^7^

The present study observed that 45 children (4.21%) with COVID-19 infection required hospital admission. This finding aligns with a study conducted in Turkey, which reported that children with rheumatic diseases did not have higher hospitalization rates for COVID-19 compared to their healthy counterparts.^8^ During the same period, the hospitalization rate for adult patients with rheumatic diseases in China was 5.29% (231 out of 4370), which is marginally higher than the rate observed in our study.^21^ In comparison, among Chinese pediatric patients in the fields of hematology and oncology—who are in a similarly immunocompromised state as our study population—8.17% (25 out of 306) required hospitalization following COVID-19 infection.^22^ This is consistent with findings from the United States, which demonstrated that symptomatic subjects with mild COVID-19 symptoms indicate minimal risk of severe or critical COVID-19 in immunosuppressed pediatric rheumatic disease patients.^10^

The pathogenesis of COVID-19 represents two distinct but overlapping phases of COVID-19 infection. The first phase is initiated by the virus itself, commencing when the infectious virus enters the respiratory tract and has the capability to infect alveolar cells through binding to angiotensin-converting enzyme 2 (ACE2) receptors, resulting in initial cold and flu-like symptoms.^23^ The elevated infection rate among children with the Omicron variant is associated with an increased binding affinity for the receptor-binding domain (RBD)-ACE2 complex of Omicron.^24,25^ One reason for the predominance of asymptomatic infections and milder disease severity among children with COVID-19 compared to adults may be related to the different immune profiles of the two. Compared to adults, the immune system of the upper respiratory mucosa in children is in a preactivated state and therefore can release interferons and control the infection early in COVID-19.^14^ In contrast, in adult COVID-19 patients, more robust adaptive immunity may instead lead to a dysregulated systemic inflammatory response resulting in multiorgan damage.^26^ Another explanation for the milder symptoms observed in children is their primary exposure to adult family members. Our data showed that family transmission was the primary mode of infection among children, accounting for 64.11% of cases, consistent with the findings of previous studies.^27^ Therefore, children may be infected with second- or third-generation viruses, potentially leading to a decrease in the pathogenicity of the strain [9]. Children infected with COVID-19 are still at risk of severe disease, the second stage of infection, the “cytokine storm”,^23^ which manifests as respiratory failure, myocarditis, shock, acute renal failure, coagulopathy, neurological involvement (encephalitis and stroke, cerebral edema, Guillain–Barre syndrome) and multisystem organ failure. While the occurrence is rare, a small percentage of children may develop a particularly severe condition called MIS-C after infection,^28^ a very concerning condition in children with COVID-19.

Our results indicated that vaccines may help reduce serious clinical outcomes among children with COVID-19. It has been reported that one of the advantages of COVID-19 vaccination is protection against long-term COVID-19 and its associated conditions, as well as a reduced risk of hospitalization.^29,30^ Our study consistently confirms that vaccination have positive impacts on children with rheumatic diseases. A study by Hu et al.^31^ showed that children who received vaccination from primary carers who had received more than two doses of a vaccine had shorter nucleic acid conversion times. Therefore, active vaccination of both eligible children and carers should be considered to reduce the risk of infection and disease in childhood cases within the family. Despite lower seropositivity rates for COVID-19 vaccination among patients with rheumatic diseases than in the general population,^32^ the European Alliance of Associations for Rheumatology (EULAR) recommends that all rheumatic patients, including those taking corticosteroids, methotrexate, bDMARDs or tDMARDs, should receive the COVID-19 vaccine.^33,34^

The results of the attribution analysis revealed that children with primary immunodeficiency had prolonged symptom durations compared to those with other rheumatic diseases. This can be attributed to their low autoimmune function and compromised ability to clear viruses. On the other hand, younger children may exhibit shorter durations of symptoms due to their limited ability to express their feelings effectively. Additionally, the shorter symptom durations observed in children treated with prednisone can be attributed to the use of glucocorticoids, which reduce the inflammatory response following an infection.

This study has several limitations. First, we did not include a control group of healthy children with COVID-19 for comparison. The children admitted to the hospital often had severe symptoms, which may have introduced selection bias, as these children are not representative of the general pediatric population. Second, the scope of symptoms evaluated was limited. The questionnaire relied on self-reporting by families, which could have resulted in incomplete information and subjective differences in symptom interpretation. Third, the questionnaire was not distributed in intensive care units, emergency departments, or other high-prevalence severe illness areas, leading to a potential underrepresentation of more severe cases of COVID-19. Furthermore, because we did not differentiate between active and inactive disease status for diseases known to have a prolonged course (e.g., juvenile idiopathic arthritis, systemic lupus erythematosus) versus monophasic diseases (e.g., Henoch-Schonlein purpura). Those with inactive disease may have experienced milder symptoms of COVID-19, might contributing to potential bias in the study findings, despite of the majority of the participants have chronic rheumatic diseases.

In summary, this study is the first to provide data on COVID-19 infection in children with rheumatic diseases in China, shedding light on the clinical and epidemiological characteristics of the disease in this demographic. The results indicate that the current wave of COVID-19 infections in this population has been mild, predominantly manifesting as upper respiratory symptoms. This study not only raises awareness regarding the health outcomes for children with rheumatic diseases who contract COVID-19 but also offers guidance on the use of medications for their primary condition. The findings highlight the importance of promoting vaccination against COVID-19 in this vulnerable population. Further studies are needed to explore the long-term effects of COVID-19 on children with rheumatic diseases.

## Supplementary information

Appendix

## Data Availability

The data is available from the corresponding author by request.
