# Supplementary material for: Clinical characteristics and outcomes of COVID-19 in pediatric patients with rheumatic diseases
Source: Pediatr Res. 2024 Oct 7;97(6):2045–55. doi: 10.1038/s41390-024-03561-1 (PMC12122361; doi:10.1038/s41390-024-03561-1)
Supplement: Supplementary file 1 — Appendix [file 41390_2024_3561_MOESM1_ESM.pdf]

Table S1 Comparison of COVID-19 vaccination with hospitalization and days of symptoms

	Group	Hospitalz -ation cases	non-hospi -talization cases	P-value	days of symptoms	P-value
COVID-19 vaccination	Not vaccinated	33	578	0.025	6.87±2.85	0.692
	Vaccinated	12	447		6.78±2.83	

Table S2 Impacts of COVID-19 vaccination doses on hospitalization rates and symptom duration

		Hospitalization			Symptoms less than 3 days			Symptoms less than 7 days		
		OR	95%CI	P-value	OR	95%CI	P-value	OR	95%CI	P-value
Unvaccination	0 dose	**Ref.**			**Ref.**			**Ref.**		
COVID-19 vaccination	one dose	1.19	0.28-3.50	0.776	0.67	0.25-2.32	0.472	1.06	0.55-1.97	0.061
	two doses	0.40	0.17-0.83	0.021*	1.13	0.64-2.03	0.682	1.17	0.89-1.55	0.159
	three doses	0.35	0.02-1.68	0.306	0.34	0.15-0.82	0.010*	1.35	0.73-2.41	0.297

* P-value is statistically significant.
